# Nano-Diamond-Enhanced Integrated Response of a Surface Plasmon Resonance Biosensor

**DOI:** 10.3390/s23115216

**Published:** 2023-05-31

**Authors:** Yu-Chun Lu, Bin-Hao Chen, Tung-Yuan Yung, Yu-Chih Tzeng, Chia-Yi Fang, Ren-Jei Chung, Po-Tuan Chen

**Affiliations:** 1 Department of Chemical Engineering and Biotechnology, National Taipei University of Technology, Taipei 10608, Taiwan; yuchun.lu.chem@gmail.com; 2 Department of Vehicle Engineering, National Taipei University of Technology, Taipei 10608, Taiwan; binhao17@ntut.edu.tw; 3 Nuclear Fuels and Materials Division, Institute of Nuclear Energy Research, Taoyuan 32546, Taiwan; romeoyung@yahoo.com; 4 Department of Power Vehicle System Engineer, Chung Cheng Institute of Technology, National Defense University, Taoyuan 33551, Taiwan; s933003@ccit.ndu.edu.tw; 5 LuminX Biotech Co., Ltd., Taipei 11503, Taiwan; cyfang@luminxbiotech.com

**Keywords:** biosensor, surface plasmon resonance, diamond nanoparticle, extraordinary optical transmission

## Abstract

Surface plasmon resonance (SPR) sensing is a real-time detection technique for measuring biomolecular interactions on gold surfaces. This study presents a novel approach using nano-diamonds (NDs) on a gold nano-slit array to obtain an extraordinary transmission (EOT) spectrum for SPR biosensing. We used anti-bovine serum albumin (anti-BSA) to bind NDs for chemical attachment to a gold nano-slit array. The covalently bound NDs shifted the EOT response depending on their concentration. The number of ND-labeled molecules attached to the gold nano-slit array was quantified from the change in the EOT spectrum. The concentration of anti-BSA in the 35 nm ND solution sample was much lower than that in the anti-BSA-only sample (approximately 1/100). With the help of 35 nm NDs, we were able to use a lower concentration of analyte in this system and obtained better signal responses. The responses of anti-BSA-linked NDs had approximately a 10-fold signal enhancement compared to anti-BSA alone. This approach has the advantage of a simple setup and microscale detection area, which makes it suitable for applications in biochip technology.

## 1. Introduction

Biosensing technology involves the detection and analysis of microchemical and biological molecule concentrations or intermolecular reactions in biological samples [1]. The structure of the biosensor contains an analysis interface on which special biomolecular receptors are fixed to sense the target molecules to be detected, as well as a transducing microsystem that converts the sensing results into other signals. Biosensors are a type of energy conversion system that can be classified as electrochemical, optical, magnetic, or piezoelectric. In recent years, optical biosensors have been actively studied owing to their high sensitivity and rapid detection ability.

Surface plasmon resonance (SPR) biosensors are optical sensors widely used to detect trace gases and biomolecules [2,3,4,5]. SPR has the advantage of not requiring fluorescence calibration of the object to be tested and can achieve the real-time detection of biological reactions, which is of great help in the research of reaction mechanisms between biomolecules [6]. SPR biological microsensing systems mainly detect reactions between biomolecules and sensor strips by measuring changes in the refractive index of the dielectric layer. In general, the conditions for SPR generation are affected by the wavelength and angle of the incident light, as well as the refractive index of the metal layer materials and dielectric layers. The dielectric layer is a biological reaction layer fixed on the metal surface. Taking the antigen–antibody reaction as an example, when the antibody fixed on the metal surface binds to the antigen in the object to be tested, the thickness and surface characteristics of the dielectric layer change. The slight change in the refractive index makes the conditions for SPR formation different from those before the reaction. From the measured results, the changes in the response of the wavelength range formed by the SPR before and after the reaction can be obtained. The response of the wavelength range is positively related to the concentration of biomolecules fixed on the metal surface after the reaction. The SPR biosensor possesses a 1 pg/mm^2^ detection limit.

Grating coupling in chip-based systems is one of the most widely used SPR setups [7]. Chip detectors with nanoarray gratings have been found to have excellent sensitivity [8,9]. In addition, a periodic metallic structure was investigated, which can be used to investigate biomolecular interactions [10,11,12]. SPR [13] and cavity modes [14] were obtained from the grating-coupled spectrum. The SPR mode is the surface plasmon excited by both sides of the slit when a transverse magnetic (TM) wave passes through the slit. The cavity mode is a gap plasma wave generated on the slit surface [15]. When these two types of surface plasmons are coupled, their spectra become observable in experiments and through simulations. Extraordinary optical transmission (EOT) has been observed in periodic subwavelength nanoholes. The EOT refers to the transmittance of light at a certain wavelength, which is particularly high in grating coupling.

The resonance response of SPR is influenced by the environmental refractive index of the structural surface according to the Bloch wave surface plasmon polariton (BW-SPP) model [16]. Changes in the refractive index of the sensor strips shift the plasmon resonance conditions, which can be detected as wavelength shifts in sensing applications [17]. Additional materials, such as nanoparticles and nanostructures with various special shapes, can be accurately fabricated and manipulated from subwavelength to nanoscale wavelengths. EOT-based techniques are suitable for miniaturization [18,19,20]. A typical approach for the improvement of SPR detection sensitivity is the labeling of analyte molecules with plasmon-active materials such as gold nanospheres and gold nanorods, which, depending on the sensing method, might increase the sensitivity by 25–100 times [21,22,23]. The development of nanofabrication methods and nanoparticle synthesis may enhance detection sensitivity. 

Nano-diamonds (NDs) have a high refractive index, *n* = 2.42; thus, labeling increases their sensitivity. Diamond is a carbon-based material with highly biocompatible and non-toxic properties that can be used in biological applications. Because of their unique chemical and optical properties, NDs have attracted considerable attention in several fields. However, few studies have discussed the use of NDs to enhance the environmental refractive index of EOT and achieve the goal of biological detection [24,25]. In this study, we used ND as an enhancement particle in a grating coupling sensor to detect wavelength shifts via the relatively high refractive index of diamond. First, the gold nanograting was fabricated. The spectrum intensity of the integrated response was enhanced in the EOT system. The ND particles were then deposited onto the substrate. Anti-bovine serum albumin (anti-BSA) was used to bind NDs for chemical attachment to a gold nano-slit array. On the substrate, the sensitivity of the wavelength shift increased because of the high refractive index of the NDs. This approach for the enhancement of sensitivity in the EOT measurement technology reached 10 times that of the signal. This investigation serves as a basis for the development of bio chips for probe–target hybridization measurements. 

## 2. Materials and Methods

### 2.1. Fabrication of the Nano-Slit Array

**Figure 1:**
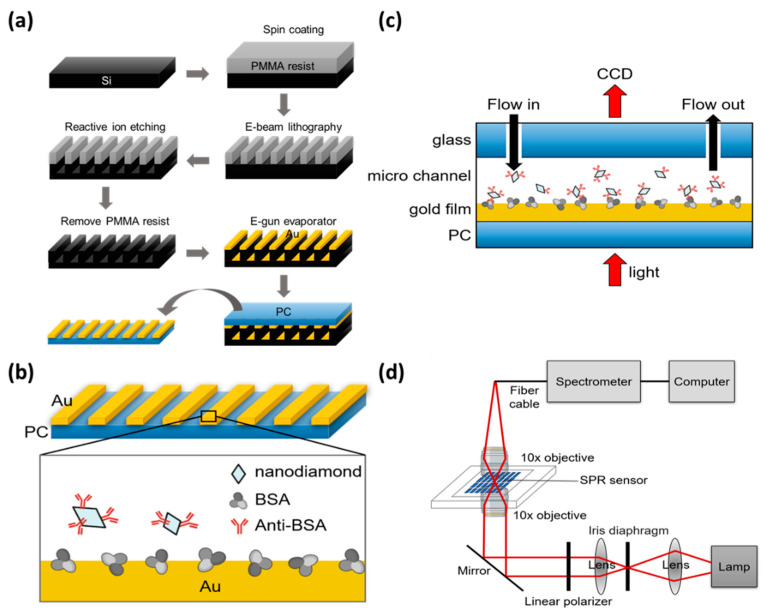
(**a**) Fabrication process of Au nano-slit arrays on a silicon substrate. (**b**) Adsorption of anti-BSA-ND particles on Au nano-slit arrays with BSA. (**c**) Light transmission experiment system and microflow channel for rinsing anti-BSA-ND solution. (**d**) Optical setup for EOT measurement.

Gold nano-slits on a silicon substrate were fabricated using electron beam lithography and a reactive ion etching method, as reported in previous studies [26,27] and shown in Figure 1a. A poly(methyl methacrylate) (PMMA) resist was spin-coated onto a 2 × 2 cm^2^ silicon film. A nano-slit pattern was drawn on the PMMA resist using an electron beam lithography system and then transferred to a silicon substrate using a reactive ion etching machine (Plasmalab 80plus, Oxford Instruments, Abingdon, UK). The power of the radio frequency electromagnetic waves in the reaction chamber was 150 W. The chamber pressure was 1 × 10^−2^ torr. The PMMA resist was then removed by washing with acetone and sputtering with ozone. The pattern was washed with deionized water in an ultrasonic bath for 20 min and dried with nitrogen. A periodic pattern of nano-slit arrays with 150 nm depth and 60 nm width was generated. 

Metallic nanostructures were fabricated on a polycarbonate (PC) substrate using a thermal annealing-assisted template stripping method [28,29]. A 50 nm thick gold film was deposited onto the nano-slit patterns by electron gun evaporation. Nanoimprinting was performed using a hot-pressing machine (Personal Arrayer 16 Microarray Spotter, CapitalBio Corp., Beijing, China). The template and the PC substrates were placed onto a hot plate heated to a temperature of 170 °C to soften the PC substrate. A gold array was printed on a PC substrate. The template and substrate were cooled and removed from the chamber. Because the gold film exhibited poor adhesion to the silicon template, the PC film was easily separated from the silicon template. Ultimately, we obtained 150 µm × 150 µm gold grating with a period of 500 nm and a slit width of 75 nm. 

### 2.2. Nano-Diamond Fabrication

Particle aggregation was the first obstacle to overcome when using ND to distribute on the surface of the substrate. Anti-BSA is a good stabilizer, and anti-BSA-coated ND nanoparticles can resist aggregation in PBS for more than several days [30]. ND was surface-functionalized by the noncovalent coating of anti-BSA [31]. Anti-BSA is a protein polymer that wraps around the outer layer of ND. Suspended 35 nm synthetic-type Ib diamond nanoparticles (Microdiamant MSY, Lengwil, Switzerland) were purified in a highly concentrated H_2_SO_4_/HNO_3_ (3:1, vol/vol) solution at 90 °C for 30 min to remove impure materials (carbon, carbon nanotubes, and graphite) on the ND surface. Deionized water was used to rinse nanoparticles several times until the diamond solution became neutral. ND particles (5 mg) were resuspended in 5 mL of phosphate-buffered saline (PBS) solution, and 4.7 mg of anti-BSA (Sigma–Aldrich, Burlington, MA, USA) was added and left at 5 °C overnight. The solution was then centrifuged to remove the uncoated anti-BSA, and the anti-BSA-ND particles were suspended in PBS for EOT measurement. This scheme is shown in Figure 1b.

### 2.3. Chemicals and Assays

We used BSA (Sigma–Aldrich, Burlington, MA, USA) and anti-BSA to investigate the biosensing capability of the ND decoration. BSA in PBS with concentrations of 0.8, 4, 20, 100, 500, and 2500 μg/mL was printed on 500 nm periods and 75 nm wide gold nano-slit arrays via a microarray printer. After drying with clean air, the gold nano-slit was sealed in a fluid channel with 0.5 mg/mL anti-BSA-ND PBS solution injection. The anti-BSA-ND particles were adsorbed on the Au nano-slit through binding with BSA.

To conduct the EOT experiment, we created a fluid channel for the sample and a control-flowing PBS solution with biological samples for detection in different environments on the chip through the channel. To manufacture the flow channel, a laser engraving machine was used to carve out the water outlet, flow channel, and sample placement area on the acrylic plate. An iron block was used to press the acrylic plate and pressurize and heat the acrylic to form an acrylic adhesive. Adhesive acrylic was installed with pipes at the inlet and outlet, and glue was used to form a seal around it, completing the reusable microflow channel, as shown in Figure 1c. 

### 2.4. Optical Setup

Figure 1d illustrates the optical setup for EOT measurement. A monochromator (Acton Research Corporation, Hsinchu, Taiwan) with 150 grooves/mm grating and blaze wavelength of 500 nm was illuminated using a white light source (Fiber-Lite MI-150, Dolan-Jenner, Boxborough, MA, USA). The light was filtered through an aperture and collimated using a lens. The incident polarization was controlled using a linear polarizer. A 10× objective lens was used to focus polarized light onto the sample. The incoming light was transmitted through the grating, collected by a second 10× objective lens, and focused onto an optical fiber. The transmitted light was projected onto a large-area charge-coupled device (CCD, 270 × S, Pixel Fly). The intensity images of the nano-slit arrays were scanned and recorded by the CCD. Real-time multispectral changes were calculated using MATLAB [32] according to the integrated response method. The area of each array was selected based on its aperture diameter. The spectra were measured using a fiber-coupled linear CCD spectrometer (Glacier X, B&W Tek, Newark, DE, USA). The NDs were mounted onto a plexiglas microfluidic channel. The refractive index sensitivities were measured by covering the samples with purified water.

## 3. Results and Discussion

### 3.1. Characterisation of Diamond Nanoparticles Coated with Anti-BSA

**Figure 2:**
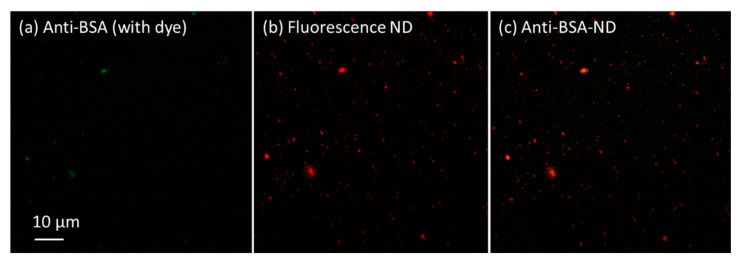
Confocal microscopy images of 100 nm fluorescence NDs and Alexa Fluor*^®^* 488 antigen dye.

The 100 nm fluorescence nano-diamonds (FNDs) were coated with anti-BSA via the above method, and fluorescent dyes (Alexa Fluor^®^ 488 Goat anti-Rabbit IgG) were added and stirred for 30 min. Confocal microscopy (Nikon) was used to acquire fluorescence images. Confocal microscopy is an optical imaging technique that utilizes point by point illumination and spatial pinhole modulation to remove scattered light from the non-focal plane of a sample. Compared to traditional imaging methods, it can improve optical resolution and visual contrast. The images can be seen in Figure 2, where the green light is the fluorescent dye, the red light is the FND, and the merged image is orange. 

Although the green light spots from the fluorescent dye do not show much detail, we can still observe some large bright spots on the left side of Figure 2a. In addition, we can see red light spots at the same position in Figure 2b, which are from the FNDs. After combining these two fluorescence images, we can clearly see orange spots in Figure 2c, indicating that the FND surface was well modified with anti-BSA. Good colocalization of FND and the dye can be seen the orange spots, which shows that the ND surface coating process worked well. This experience was obtained with a concentration series of anti-BSA and FNDs. We did not find a large difference in the results and chose 5 mg of ND particles in 5 mL of PBS solution with 4.7 mg of anti-BSA added as the sample. There was no difference in test results regarding whether FND or ND could enhance the SPR signal. Therefore, we used ND for the following experiments.

**Figure 3:**
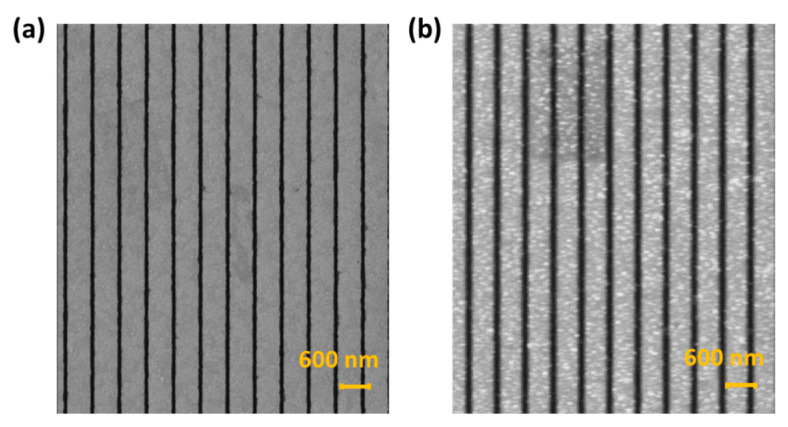
Scanning electron microscope (SEM) images of (**a**) template-stripped nano-slit arrays and (**b**) diamond nanoparticles on the surface of the nano-slits.

Figure 3a shows a scanning electron microscopy (SEM) image of the nano-slit arrays fabricated using the thermal annealing-assisted template stripping method. We can clearly see the structure of the nano-slits with a 500 nm wide band and 75 nm wide slits. Figure 3b shows an SEM image of the anti-BSA-ND nanoparticles on the surface of the nano-slits. The SEM images demonstrate that the NDs were successfully deposited onto the nano-slit. As the concentration of NDs increased, more ND particles were deposited on the surface, as expected. Nevertheless, we chose a ND concentration of 2500 ug/mL for demonstration.

### 3.2. Integrated Response Method

Fano-type resonances are based on the coupling of periodic nanostructures with incoming waves [33]. Periodic gold nanostructures with NDs exhibit complex resonant behavior in the transmission spectrum owing to the coupling of the localized plasmon resonance in the subwavelength apertures and the BW-SPP [34] on the periodic surface. By summing all transmission changes in the spectral image, the signal-to-noise ratio of the system is improved, and the sensing capability can be enhanced. The signal generated from the SPR spectrum corresponds to the injection of light of a specific wavelength into the grating chip. Because of the change in the environmental refractive index, response signals of that wavelength were obtained.

(1) $$S_{I}(\lambda )=\frac{R}{∆n}$$

(2) $$R=\left|\left.\frac{\left(I_{X}-I_{0}\right)}{I_{0}}\right|\times 100\%\right.$$

*S_I_* is the environmental refractive index sensitivity, which is the function of the incident light wavelength, λ, and Δ*n* is the change in the refractive index. *R* is the percentage change in the intensity response value of the chip spectrum, *I_X_* is the intensity of the light transmitted through the chip with a certain refractive index, and *I*_0_ is the intensity of the transition light through a reference medium (PBS). Thus, changes in the surface environment may influence the resonance wavelength of abnormal light penetration. The sensitivity of surface plasmon waves to modifications of the environmental refractive index can be used in biological or chemical detection experiments. Therefore, the ND particles were introduced onto the *Si* substrate with a gold array.

However, this conventional method is insufficient to cope with general measurement experiments. This is because it takes considerable time to select the wavelength with the largest intensity change rate in the transmission spectrum from the wide range of wavelengths of the scanning light. Moreover, the wavelength with the highest change will not be the same because of small differences in chip fabrication. To improve the signal-to-noise ratio and sensing capability of the EOT system, integration of the total response within all wavelengths was applied. This method has been demonstrated for quasi-3D plasmonic crystals and increases the detection limit [35]. The intensity sensitivity is defined as follows:

(3) $$R=\int \left|\frac{T\left(n,\lambda \right)-T(n_{0},\lambda )}{T(n_{0},\lambda )}\right|d\lambda ,$$

where *T(n,λ)* is the transmission spectrum under a different external refractive index *n*, and *n*_0_ is the referenced refractive index (*n*_0_ = 1.3330 for pure water). Using this calculation method, even if the spectrum changes only slightly in media with different refractive indices, it can significantly enhance the signal performance and is not limited to observing the intensity change of a single wavelength. In this study, the light separated by a single light meter was discontinuous; therefore, the formula was slightly modified and used in practice as follows:

(4) $$R=\sum_{\lambda_{2}}^{\lambda_{1}}\left|\frac{T\left(n,\lambda \right)-T(n_{0},\lambda )}{T(n_{0},\lambda )}\right|\Delta \lambda ,$$

where *λ*_1_ and *λ*_2_ are the integrated wavelength ranges, and Δ*λ* is the resolution of the spectrometer.

### 3.3. Anti-BSA vs. Anti-BSA-ND EOT Results

After the anti-BSA-ND particles were prepared, microarrays with different BSA concentrations were used to study the wavelength shift of the EOT system. We injected anti-BSA-only and anti-BSA-ND solutions into the EOT system and recorded the wavelength shifts over an average of 100 s values. Initially, the microchip was washed with PBS solution to remove unbonded BSA from the microarray, and anti-BSA and anti-BSA-ND solutions were injected to measure the signal changes in the reaction. The microchip was then washed again with PBS solution to remove unreacted antigen and anti-BSA-ND particles. 

**Figure 4:**
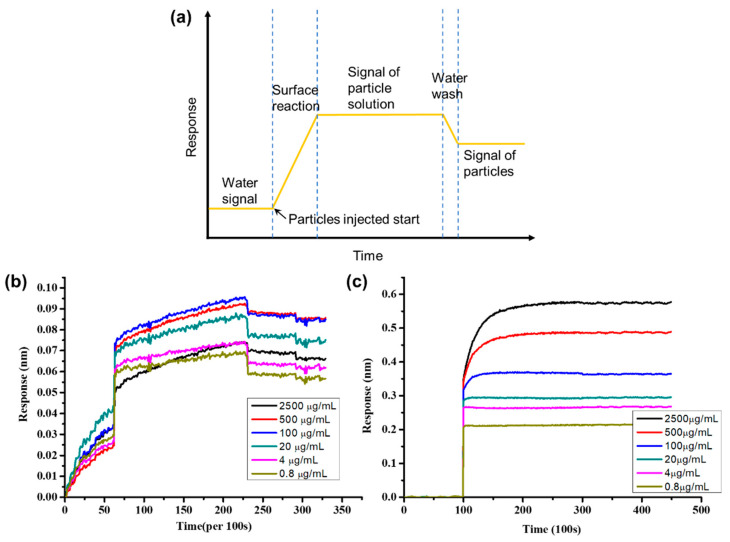
(**a**) Scheme for measured integrated responses as a function of the interaction time for various concentrations of PBS. (**b**) Integrated response with variation of the anti-BSA-only concentration. (**c**) Integrated response with variation of the anti-BSA-ND solution.

Combining 2-D spectral images and the spectral integration method, we demonstrate real-time and multiple detections of 35 nm NDs. In the experiment, the BSA in PBS solution of various concentrations (0.8, 4, 20, 100, 500, and 2500 μg/mL) was spotted on the nano-slit array using a microarray machine. The chip was then mounted onto a microfluidic device. Figure 4 shows the measured integrated responses as a function of the interaction time for different BSA spots. Figure 4a shows the scheme of this experiment. Initially, pure PBS solution was injected into the system for 2 to 3 h to remove unreacted BSA salt of the chip, and then 500 ug/mL of anti-BSA/anti-BSA-ND solution was injected for 4 to 6 h to obtain response signals. Finally, PBS was injected into the system again for 3 h to wash it out. The anti-BSA response is shown in Figure 4b, and the anti-BSA only solution response is shown in Figure 4c. We can clearly see that the anti-BSA-ND response is much stronger than that of the anti-BSA-only solution; the signals of anti-BSA-only solution after washing are too small to find. Anti-BSA only solution wavelength shifts are from 0.02–0.06 nm (Figure 4b), much smaller than the anti-BSA-ND wavelength shift of 0.2–0.6 nm (Figure 4c). On the other hand, anti-BSA-ND can provide 10 times signal enhancement compared with the anti-BSA solution. 

**Figure 5:**
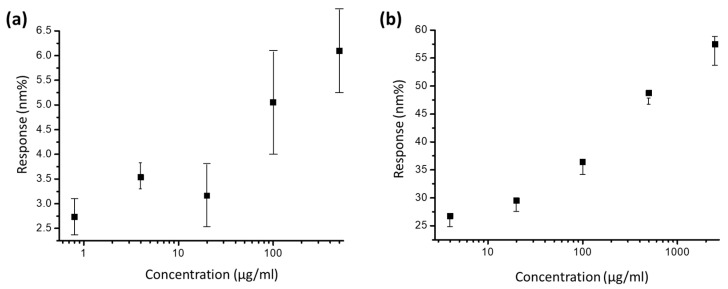
Quantization of the results for integrated response with variation of (**a**) anti-BSA-only concentration and (**b**) anti-BSA-ND solution.

The quantization of the results for the measurements in Figure 5b indicates that the signal response of anti-BSA-ND ranges from ~25 to 55 nm%. The anti-BSA and BSA interaction signal changes range from ~2.75 to 6 nm% (Figure 5a) in the same EOT integration system, which is one-tenth smaller than the anti-BSA-ND and BSA interactions. The detection limit of the 35 nm anti-BSA-ND sample is 7.86 µg/mL, which is also better than the detection limit of the anti-BSA sample (12.5 µg/mL). As the concentration of 2500 μg/mL is too high, resulting in many molecules remaining on the chip surface during measurement, saturation occurs, resulting in much lower measurement results than expected. Therefore, the figure displays non-linear trends. To date, ND particles have been used as protein substrates to enhance EOT integration.

**Figure 6:**
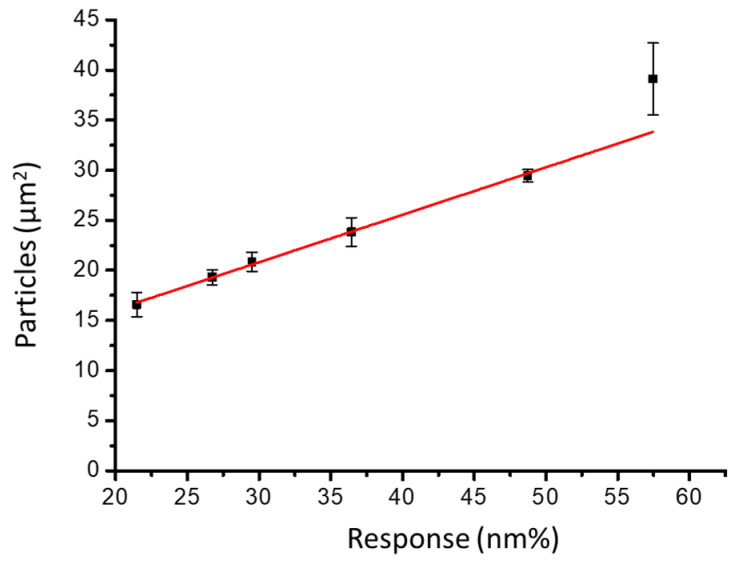
Response change as a function of ND size distribution on the gold nano-slit surface.

After washing, the nano-slit array was scanned using SEM. The SEM image shows an approximately equal distribution of attached NDs over the microarray area. The ND particle distribution was observed using SEM images, and the average particle size was calculated. We calculated the response (nm%) versus ND particle distribution on the gold nano-slits (particles/μm^2^). Figure 6 shows the response change as a function of the particle density. A linear correlation exists between the response change percentage (nm%) and particle density (particles/μm^2^). The slope is 0.47344 ± 0.02708 (particles/(μm^2^ nm%)), and the slope intercept is 6.6971 ± 1.02813 (particles/μm^2^). The current noise in the system is 0.13 nm%. Therefore, the detection limit of the 2D spectral image method is 0.06157 ± 0.00352 particles/μm^2^.

## 4. Conclusions

The development of SPR biosensors aims to improve sensitivity for achieving the goal of miniaturization of the system. In this study, we synthesized a chip-type SPR biosensor with NDs, which is more convenient and increases the accuracy of measurement through optical path detection and microchannel testing. In particular, we demonstrate the use of NDs to enhance the sensitivity of biosensing based on EOT spectrum detection. 

The fabrication of the wafer involved the use of an electron beam microimaging system and reactive ion etching equipment to produce high-quality and mass-produced biosensor wafers with a nanoslot-structured silicon master mold coated with gold that was then transferred to a transparent PC film. The effect of ND enhancement depends on the refractive index of the environment. The change in the refractive index of the gold array by the NDs changes the EOT spectra. We used NDs covalently bound to the gold nano-slit array for these measurements. Therefore, a new method for the chemical attachment of NDs to gold surfaces was developed. We demonstrated that NDs are a good material for integrating EOT system signal enhancement. For the experiments with anti-BSA and BSA, a 10-fold signal enhancement was observed from ~2.5–6 nm% to ~25–55 nm%, and the detection limit increased from 12.5 µg/mL to 7.86 µg/mL. We hope this paper will inspire more studies of carbon-based materials for SPR detection.

## Data Availability

Not applicable.
